# Evaluation of honeys and bee products quality based on their mineral composition using multivariate techniques

**DOI:** 10.1007/s10661-012-2847-y

**Published:** 2012-08-29

**Authors:** Małgorzata Grembecka, Piotr Szefer

**Affiliations:** Department of Food Sciences, Medical University of Gdańsk, Al. Gen. J. Hallera 107, 80-416 Gdansk, Poland

**Keywords:** Honeys, Metals, AAS, ANOVA, Factor analysis, Cluster analysis

## Abstract

The aim of this investigation was to estimate honeys and bee products quality in view of their mineral composition using multivariate techniques. Fourteen elements (Ca, Mg, K, Na, P, Co, Mn, Fe, Cr, Ni, Zn, Cu, Cd, and Pb) were determined in 66 honeys and bee products from different places of Poland and Europe and various botanical origins. The total metals contents were analyzed by flame atomic absorption spectrometry using deuterium-background correction after wet digestion with nitric acid in an automatic microwave digestion system. Phosphorus was determined in the form of phosphomolybdate by a spectrophotometric method. Reliability of the procedure was checked by analysis of the certified reference materials tea (NCS DC 73351) and cabbage (IAEA-359). The analytical data indicated a good level of quality of honeys, especially with regard to the concentration of toxic trace elements, such as Cd and Pb. Results were submitted to multivariate analysis, including such techniques as factor and cluster analyses in order to evaluate the existence of data patterns and the possibility of classification of honeys from different botanical origins according to their mineral content. The nine metals determined were considered as chemical descriptors of each sample. There was a significant influence of the botanical and geographical provenance as well as technological processing on the elemental composition of honeys.

## Introduction

Honey is the food product that the domesticated bees (*Apis mellifera* L.) produce and transform from the nectar of flowers or from the sugar secretions from the leaves of arboreal essence. Besides being healthy and easy to digest, this natural product is full of carbohydrates, vitamins, minerals, and enzymes (Alvarez-Suarez et al. 2010; Hernández et al. 2005). Other bee product, propolis, is actually a complex mixture of resins and other substances that honeybees use to seal the hive and make it safe from bacteria and other microorganisms (Xu et al. 2009). Honeybees make propolis by combining plant resins with their own secretions. Its composition varies considerably from region to region as well as along with the vegetation. Similarly to honey, it is also believed to promote heart health, strengthen the immune system, and reduce the chances of viral diseases (Xu et al. 2009).

Honeys can be found in all types of colors and flavors from nearly colorless to dark brown, and its flavor varies from delectably mild to distinctively bold since both the flavor and color are directly influenced by the type of nectar gathered by the bees from various floral sources. Usually a lighter color will indicate a milder flavor, while darker honey is customarily more robust and contains more minerals. Varietal honey is rarely 100 % of any one type of flower nectar but a blend with a predominance of one type of flower forage. In addition to being a natural nutritive sweetener, research also indicates that honey’s unique composition makes it useful as an antimicrobial agent and antioxidant. Honey also reduces skin inflammation, edema, and exudation as well as promotes wound healing, diminishes scar size, and stimulates tissue regeneration (Alvarez-Suarez et al. 2010). It has been also found that it ameliorates cardiovascular risk factors as well as being a potent inhibitor of *Helicobacter pylori*. What is more, honeys express antimutagenic activity against bladder cancer and mammary carcinoma (Alvarez-Suarez et al. 2010). The health benefits of honey depend on its quality that is strongly associated with its chemical composition and floral origin.

Food quality requires the control of nutritional value, sensorial properties, authenticity, and safety. Honeys come from a wide range of geographical areas and may have varied chemical and organoleptic properties. Therefore, it is very important to have methods to characterize different honey varieties. Their common characteristics is moisture content below 20 %, a reducing sugar content of 60–65 %, and a bulk sucrose content of 5–10 % (Alvarez-Suarez et al. 2010; Hernández et al. 2005). However, it is possible to find parameters that could differentiate honeys and one of such criteria is their metal content (Hernandez et al. 2005; Tuzen et al. 2007). Metals concentration ranges from about 0.04 % in light color honeys to 0.2 % in some dark honeys, and protein content of honey is usually lower than 0.5 % (Alvarez-Suarez et al. 2010; Fernàndez-Torres et al. 2005).

Advanced statistical chemometric techniques are very effective in analytical evaluation of food quality. Based on the mineral composition data, it is possible to record the influence of elements on the distribution of particular object samples and classify food products according to their country of origin, type, and genetic classification (Szefer 2007). Several authors have applied chemometrical procedures on elemental composition data in order to classify honeys in view of their botanical and geographical provenance (Chudzinska and Baralkiewicz 2010; Devillers et al. 2002; Fernàndez-Torres et al. 2005; Hernández et al. 2005; Latorre et al. 1999; Pisani et al. 2008).

The aim of the present investigations was to analyze and compare concentrations of macro- and microelements and toxic metals in 66 brands of commercially available honeys and bee products from different regions of Poland and Italy (Table 1) obtained from the local market in Poland. Moreover, we wanted to define honey quality with regard to several toxic elements such as lead and cadmium. Due to relatively low cost and quite good analytical performance, flame atomic absorption spectrometry (FAAS) has been used for analyses. For the classification and discrimination between the different types of honey, factor and cluster analyses were carried out. Results of the analyses highlight the potential of the use of elemental composition for the discrimination and classification of honey and bee products in view of their botanical provenance, type, and level of technological processing.

**Table 1:** Characteristics of the analyzed products

Product	Details	Product	Details
Honeys			
Acacia honey	Certified origin, North Eastern Poland	Honeydew honey	Certified origin, South Eastern Poland
Acacia honey	Certified origin, North Eastern Poland	Multifloral honey	Certified origin, Northern Poland
Acacia honey	Certified origin, central Poland	Multifloral honey	Certified origin, central Poland
Acacia honey	Certified origin, South Eastern Poland	Multifloral honey	Certified origin, central Poland
Acacia honey	Certified origin, South Eastern Poland	Multifloral honey	Certified origin, North Eastern Poland
Eucalyptus honey	Certified origin, Italy	Multifloral honey	Certified origin, central Poland
Buckwheat honey	Certified origin, South Eastern Poland	Multifloral honey	Certified origin, central Poland
Buckwheat honey	Certified origin, central Poland	Multifloral honey	Certified origin, Italy
Buckwheat honey	Certified origin, North Eastern Poland	Heather honey	Certified origin, North Eastern Poland
Buckwheat honey	Certified origin, central Poland	Heather honey	Certified origin, Southern Poland
Buckwheat honey	Certified origin, South Eastern Poland	Artificial honey	Produced in Poland
Buckwheat honey	Certified origin, central Poland	Artificial honey	Produced in Poland
Apple honey	Certified origin, Italy	Syrup-feed honeys	
Chestnut honey	Certified origin, Italy	Aloe syrup-feed honey	Produced in Poland
Lime honey	Certified origin, South Eastern Poland	Chokeberry syrup-feed honey	Produced in Poland
Lime honey	Certified origin, central Poland	Crataegus syrup-feed honey	Produced in Poland
Lime honey	Certified origin, Southern Poland	Stinging nettle syrup-feed honey	Produced in Poland
Lime honey	Certified origin, central Poland	Pine syrup-feed honey	Produced in Poland
Lime honey	Certified origin, North Eastern Poland	Syrup-feed honey	Produced in Poland
Lime honey	Certified origin, central Poland	Honeys with natural additives	
Lime honey	Certified origin, North Eastern Poland	Bee honey with chokeberry	Produced in Poland
Dandelion honey	Certified origin, North Eastern Poland	Bee honey with cinnamon	Produced in Poland
Orange honey	Certified origin, Italy	Bee honey with pollen	Produced in Poland
Rape honey	Certified origin, Northern Poland	Royal jelly in honey	Produced in Poland
Rape honey	Certified origin, central Poland	Propolis in honey	Produced in Poland
Rape honey	Certified origin, central Poland	Bee products	
Rape honey	Certified origin, Northern Poland	Propolis	Produced in Poland
Rape honey	Certified origin, central Poland	Propolis	Produced in Poland
Honeydew honey	Certified origin, South Eastern Poland	Bee pollen	Produced in Poland
Honeydew honey	Certified origin, North Eastern Poland	Bee pollen	Produced in Poland
Honeydew honey	Certified origin, central Poland	Bee pollen	Produced in Poland
Honeydew honey	Certified origin, North Eastern Poland	Bee pollen	Produced in Poland
Honeydew honey	Certified origin, North Eastern Poland		

## Materials and methods

### Honey and bee products samples

Honeys samples to be analyzed were purchased from the local market in Gdańsk (Poland) as well as from beekeeper’s shops throughout the country in 2004 and 2005. The samples represented the most common types of honey readily available to consumers in Poland. The honeys analyzed were classified according to the producer statement on the label. Products included natural honeys of different botanical origins (acacia, buckwheat, apple, chestnut, lime, dandelion, orange, rape, honeydew, multifloral, and heather) as well as syrup-feed honeys, honeys with natural additives such as chokeberry, cinnamon, pollen and propolis, and other bee products, including bee pollen and propolis. There were also analyzed samples of inverted sugar syrup which is commercialized in Poland under the name artificial honey. It has an appearance similar to honey and is often used as a substitute for people who do not eat honey. It consists of glucose and fructose syrup produced by inversion, which has been blended with the original sucrose syrup in a proportion that creates a thick mixture which does not crystallize. In total, 66 products (198 subsamples) were analyzed in triplicate for macroelements (Mg, Ca, K, Na, and P), microelements (Zn, Cu, Fe, Cr, Co, Ni, and Mn), and toxic elements (Pb and Cd). All analyzed products are characterized in Table 1.

### Sample preparation and analysis

Three replicates of 1-g (±0.0001 g) samples were treated with 9 ml 65 % HNO_3_ (Suprapur® Merck) and then digested in an automatic microwave digestion system (MLS 1200M) according to the following steps: I, 250 W, 48 s; II, 0 W, 48 s; III, 250 W, 6 min 24 s; IV, 400 W, 4 min; V, 650 W, 4 min. The steps are described in detail in the operation manual. Every microwave digestion cycle consisted of five food samples and one blank sample (9 ml 65 % HNO_3_). After digestion, the vessels were cooled at room temperature. Every digested sample was dissolved up to 10 ml with deionized water.

The concentrations of elements (Mg, Ca, K, Na, Zn, Cu, Fe, Cr, Co, Ni, Mn, Pb, and Cd) were determined in an air–acetylene flame by AAS method using deuterium-background correction. A Philips PU-9100 model atomic absorption spectrometer was used for metal analyses. The FAAS conditions are described in the operation manual. In the case of Na and K determinations, Cs was added to samples and standards as an ionization buffer at a concentration of 0.2 % w/v, and in the case of Ca and Mg measurements, La was used as a releasing agent at a concentration of 0.4 % w/v. Phosphorus was determined in the form of phosphomolybdate by spectrophotometric method (Official Methods of Analysis of AOAC International 2002).

Nickel, Co, Cr, Pb, and Cd concentrations in the samples analyzed were under the detection limits of the method applied, i.e., 0.02, 0.01, 0.02, 0.01, and 0.003 mg 100 g^−1^. The detection limit was established according to Konieczka and Namieśnik (2009), i.e., LD = blank mean + 3SD. The reliability of the method was tested with certified standard reference materials including tea (NCS DC 73351) and cabbage (IAEA-359). The recoveries obtained for the reference materials varied between 84.5 % and 103 %, and precisions were 0.13–13 %.

### Estimation of recommended dietary intake and provisional tolerable monthly intake

The daily mineral intake (in percent) through consumption of 25 g (one tablespoon) of the products analyzed was calculated as DMI = *C* × 100/RDA, where *C* is element concentration (in milligrams) in 25 g of product and RDA is according to the National Polish Food and Nutrition Institute (Jarosz and Bułhak-Jachymczyk 2008) or American data (Dietary Reference Intakes 2004).

In accordance with recommendation of FAO/WHO (WHO 2010a, b), a provisional tolerable monthly intake (PTMI) for Cd amounted to 25 μg/kg of body weight for adult, i.e., 490 μg monthly for 70 kg person. At the 73rd FAO/WHO Meeting (WHO 2010b), the Committee concluded that the PTWI for Pb could no longer be considered health protective and withdrew it. Furthermore, as the dose–response analyses did not provide any indication of a threshold for the key adverse effects of Pb, the Committee concluded that it was not possible to establish a new PTWI that would be health protective (WHO 2010b).

### Statistics

Spearman’s rank correlation analysis, ANOVA Kruskal–Wallis test, factor analysis (FA), and cluster analysis (CA) of the data obtained were performed using STATISTICA 8.0 for Windows (Copyright^©^ StatSoft, Inc. 1984–2007). Before the chemometric analysis, the selected variables were tested for normality. In all cases, they did not follow the normal distribution according to Shapiro–Wilk and the Kolmogorov–Smirnov tests (Brereton 2003; Szefer 2007). Therefore, non-parametric procedures were adapted in our analyses. Prior the chemometric processing, the data matrix were autoscaled. FA was performed on raw data sets concerning honeys and bee products samples. The data matrix was established using the elements as columns and analyzed products as rows. Each product’s arithmetic mean value of three subsamples was taken into consideration; therefore, 38 natural honeys and 50 natural, syrup-feed, artificial, and with natural additives honeys accounted for the final data matrix. Elements such as Pb, Cd, Cr, Ni, and Co had to be eliminated from the data set because of their too low levels; therefore, nine loadings (Ca, Mg, Na, K, P, Zn, Cu, Fe, and Mn) constituted the ultimate data matrix. All elements proved to have great contribution to samples differentiation (Tables 2 and 3). The cut-off loading value to determine which elements will be used at the clustering stage was set at the level >0.70. CA, similarly to FA, was also performed on raw data sets concerning honeys and bee products samples. The best results of CA analysis were obtained by applying the Ward method as a way of calculating of cluster distances, as well as Euclidean distance as a measure of distance between analyzed samples.

**Table 2:** Factor loadings for elements analyzed in honeys of different botanical families

	Factor 1	Factor 2
Ca	−0.193354	0.330832
Mg	−0.707836	−0.077388
Na	−0.273910	0.763634
K	−0.845422	0.295266
P	−0.720763	−0.029373
Zn	0.000804	0.403728
Cu	−0.872018	−0.345827
Fe	−0.110214	−0.584865
Mn	−0.770736	−0.104390

**Table 3:** Factor loadings for elements analyzed in different types of honeys

	Factor 1	Factor 2
Ca	0.014845	0.551394
Mg	−0.618603	0.165681
Na	0.161171	0.677685
K	−0.841946	0.183504
P	−0.767338	0.041556
Zn	−0.161139	−0.218379
Cu	−0.876076	−0.056267
Fe	−0.201850	−0.623420
Mn	−0.780438	0.049780

## Results and discussion

### Element concentrations in honeys

Data of the elements analyzed in honeys and bee products are listed in Tables 4 and 5. The minerals concentrations in the samples are characterized by arithmetic mean value, the corresponding standard deviation (SD), and ranges for wet weight basis.

**Table 4:** Concentration of the macroelements studied in honeys and bee products in mg 100 g^−1^ wet weight ($\overline x$±SD, range)

Product	*N*	*n*	Ca	Mg	Na	K	P
Honeys							
Acacia honeys	5	15	4.88 ± 1.68	1.01 ± 0.41	1.30 ± 1.67	16.6 ± 2.86	14.0 ± 9.91
			2.86–6.92	0.65–1.4	0.38–4.28	12.7–19.6	7.14–28.6
Eucalyptus honey	1	3	5.28 ± 0.50	1.76 ± 0.21	5.63 ± 0.07	45.3 ± 1.33	57.1 ± 3.61
			4.81–5.80	1.61–1.91	5.58–5.68	44.5–46.9	53.5–60.7
Buckwheat honeys	6	19	3.37 ± 1.24	1.66 ± 0.38	0.98 ± 0.21	32.2. ± 9.55	69.6 ± 18.2
			1.95–5.50	1.05–2.00	0.57–1.15	19.7–43.7	47.6–92.7
Apple honey	1	3	5.70 ± 0.43	14.5 ± 0.61	2.39 ± 0.01	73.6 ± 0.58	48.8 ± 2.05
			5.40–6.01	14.0–15.2	2.38–2.40	72.9–74.1	46.5–50.1
Chestnut honey	1	3	5.50 ± 0.13	4.93 ± 0.05	0.78 ± 0.04	70.9 ± 0.46	14.3 ± 0.01
			5.40–5.59	4.89–4.99	0.75–0.82	70.6–71.4	14.3–14.3
Lime honeys	7	21	3.97 ± 0.84	1.60 ± 0.63	1.89 ± 1.38	39.3 ± 12.6	13.0 ± 7.48
			2.55–4.80	0.73–2.70	0.92–4.76	22.4–52.8	3.58–23.8
Dandelion honey	1	3	6.11 ± 0.29	0.90 ± 0.001	7.41 ± 0.23	51.1 ± 1.62	7.15 ± 0.01
			5.90–6.31	0.90–0.90	7.25–7.57	49.2–52.1	7.14–7.16
Orange honey	1	3	2.76 ± 0.07	0.55 ± 0.07	0.95 ± 0.01	16.7 ± 0.85	51.2 ± 5.56
			2.71–2.81	0.50–0.60	0.94–0.96	15.8–17.4	46.4–57.3
Rape honeys	5	15	5.13 ± 1.66	1.89 ± 0.83	1.12 ± 0.83	19.0 ± 17.2	7.51 ± 5.71
			2.21–6.24	0.95–3.21	0.70–2.60	8.48–49.4	3.57–16.1
Honeydew honeys	6	18	5.34 ± 1.63	4.52 ± 1.42	2.00 ± 1.38	62.1 ± 4.98	61.0 ± 23.2
			2.27–7.11	2.70–6.61	0.68–3.68	52.5–66.1	23.8–85.8
Multifloral honeys	7	21	3.89 ± 0.57	1.55 ± 0.35	2.62 ± 2.51	34.4 ± 17.6	37.4 ± 8.72
			2.80–4.45	1.20–2.25	0.49–5.82	10.6–55.6	27.4–45.3
Heather honeys	2	6	7.28 ± 1.87	1.65 ± 0.14	5.55 ± 5.09	53.2 ± 8.27	57.7 ± 9.19
			5.95–8.60	1.55–1.75	1.95–9.15	47.3–59	51.2–64.2
Artificial honeys	2	6	6.54 ± 0.04	0.28 ± 0.11	13.1 ± 5.50	1.20 ± 0.16	3.57 ± 0.00
			6.51–6.57	0.20–0.35	9.22–17.0	1.11–1.34	3.57–3.57
Syrup-feed honeys							
Aloe syrup-feed honey	1	3	7.32 ± 0.35	1.57 ± 0.15	1.00 ± 0.03	11.1 ± 1.05	7.14 ± 0.01
			7.03–7.70	1.41–1.70	0.98–1.02	10.0–12.1	7.14–7.15
Chokeberry syrup-feed honey	1	3	4.35 ± 0.22	2.80 ± 0.00	1.72 ± 0.02	22.3 ± 0.60	7.14 ± 0.01
			4.20–4.51	2.80–2.81	1.71–1.73	21.8–23.0	7.13–7.15
Crataegus syrup-feed honey	1	3	4.30 ± 0.28	2.65 ± 0.07	0.60 ± 0.03	31.7 ± 0.64	29.7 ± 2.09
			4.10–4.50	2.60–2.70	0.57–0.62	31.2–32.4	28.5–32.1
Stinging nettle syrup-feed honey	1	3	7.56 ± 0.64	2.47 ± 0.06	1.74 ± 0.10	25.7 ± 0.21	27.4 ± 2.03
			7.10–8.01	2.40–2.50	1.63–1.82	25.5–25.9	25.0–28.6
Pine syrup-feed honey	1	3	3.50 ± 0.004	1.77 ± 0.15	0.89 ± 0.07	17.2 ± 0.21	27.4 ± 2.01
			3.50–3.51	1.60–1.90	0.84–0.94	17.1–17.4	25.1–28.6
Syrup-feed honey	1	3	2.25 ± 0.23	2.55 ± 0.34	0.85 ± 0.07	25.6 ± 0.63	14.3 ± 0.01
			2.09–2.41	2.31–2.79	0.81–0.93	25.2–26.3	14.3–14.3
Honeys with natural additives							
Bee honey with chokeberry	1	3	6.90 ± 0.30	5.91 ± 0.29	1.36 ± 0.04	31.1 ± 1.33	23.8 ± 2.07
			6.69–7.11	5.71–6.12	1.33–1.39	30.1–32.6	21.4–25.0
Bee honey with cinnamon	1	3	12.9 ± 0.97	2.93 ± 0.22	1.38 ± 0.06	33.0 ± 0.62	23.2 ± 2.57
			12.1–14.0	2.80–3.19	1.32–1.45	32.4–33.6	21.4–25.1
Bee honey with pollen	1	3	6.30 ± 0.70	5.80 ± 0.14	1.50 ± 0.10	42.7 ± 0.39	76.2 ± 2.07
			5.80–6.80	5.70–5.90	1.43–1.57	42.3–43.1	74.9–78.6
Royal jelly in honey	1	3	7.34 ± 0.32	2.00 ± 0.10	0.72 ± 0.01	17.9 ± 0.27	40.5 ± 2.07
			7.11–7.71	1.90–2.10	0.71–0.73	17.7–18.2	39.3–42.9
Propolis in honey	1	3	17.3 ± 0.42	15.9 ± 0.11	2.43 ± 0.002	54.2 ± 0.60	108 ± 2.03
			17.0–17.6	15.8–16.0	2.42–2.43	53.7–54.8	107–111
Acacia honey with almonds^a^	1	3	3.47 ± 0.23	2.20 ± 0.10	0.62 ± 0.01	19.8 ± 0.07	25.0 ± 0.02
			3.20–3.61	2.10–2.31	0.61–0.62	19.7–19.9	25.0–25.0
Acacia honey with peanuts^a^	1	3	2.10 ± 0.14	4.27 ± 0.30	0.59 ± 0.01	48.7 ± 0.18	46.4 ± 0.08
			2.00–2.20	4.00–4.60	0.58–0.59	48.6–49.0	46.4–46.5
Bee products							
Propolis	2	6	78.0 ± 10.3	25.3 ± 0.35	9.23 ± 7.59	49.9 ± 2.12	573 ± 50.9
			70.7–85.2	25.0–25.5	3.86–14.6	48.4–51.4	537–609
Bee pollen	4	12	95.1 ± 13.8	77.4 ± 20.4	5.86 ± 5.33	70.0 ± 0.53	659 ± 46.3
			81.4–111	48.9–92.4	2.35–13.8	69.3–70.4	611–722
Other							
Almonds^a^	1	3	160 ± 9.99	136 ± 1.93	0.51 ± 0.01	69.0 ± 0.54	91.6 ± 5.46
			151–171	134–138	0.50–0.52	68.4–69.5	85.7–96.4
Peanuts^a^	1	3	23.1 ± 2.30	105 ± 8.16	1.43 ± 0.02	68.6 ± 1.27	107 ± 5.10
			21.4–24.7	99.2–111	1.41–1.44	67.7–69.5	104–111

*N* number of products, *n* number of analytical subsamples

^a^Honey and its additives were analyzed separately

**Table 5:** Concentration of the microelements and toxic metals studied in honeys and bee products in milligrams per 100 g of wet weight ($\overline x$ ± SD, range)

Product	*N*	*n*	Zn	Cu	Fe	Mn	Cr	Ni	Co	Pb	Cd
Honeys											
Acacia honeys	3	15	0.41 ± 0.39	0.01 ± 0.00	0.12 ± 0.11	0.05 ± 0.03	ND	0.03 ± 0.00	0.01 ± 0.01	ND	ND
			0.02–0.95	0.01–0.02	0.04–0.29	0.01–0.07		<0.02–0.03	<0.01–0.02		
Eucalyptus honey	1	3	0.08 ± 0.01	0.02 ± 0.001	0.11 ± 0.004	0.10 ± 0.01	0.03 ± 0.001	0.03 ± 0.003	ND	ND	ND
			0.07–0.08	0.01–0.02	0.10–0.11	0.09–0.11	0.03–0.03	0.03–0.04			
Buckwheat honeys	6	19	0.38 ± 0.24	0.07 ± 0.02	0.67 ± 1.00	0.47 ± 0.15	ND	0.05 ± 0.02	0.01 ± 0.01	ND	ND
			0.08–0.74	0.05–0.09	0.06–2.64	0.33–0.70		<0.02–0.06	<0.01–0.02		
Apple honey	1	3	0.11 ± 0.01	0.22 ± 0.01	0.57 ± 0.02	0.12 ± 0.01	ND	0.03 ± 0.002	0.01 ± 0.001	ND	ND
			0.10–0.12	0.21–0.23	0.55–0.59	0.12–0.13		0.03–0.03	0.01–0.01		
Chestnut honey	1	3	0.07 ± 0.003	0.06 ± 0.003	0.14 ± 0.001	0.08 ± 0.004	0.05 ± 0.004	ND	<0.01	ND	ND
			0.07–0.07	0.06–0.07	0.14–0.14	0.07–0.08	0.04–0.05				
Lime honeys	7	21	0.84 ± 0.54	0.03 ± 0.01	0.25 ± 0.18	0.11 ± 0.07	0.02 ± 0.02	0.04 ± 0.04	0.02 ± 0.01	ND	ND
			0.05–1.69	0.01–0.05	0.04–0.30	0.05–0.22	<0.02–0.04	<0.02–0.08	<0.01–0.02		
Dandelion honey	1	3	0.65 ± 0.04	0.03 ± 0.003	0.08 ± 0.003	0.09 ± 0.01	ND	0.03 ± 0.003	0.03 ± 0.002	ND	ND
			0.62–0.68	0.03–0.03	0.08–0.09	0.08–0.10		0.03–0.03	0.03–0.03		
Orange honey	1	3	0.05 ± 0.001	0.01 ± 0.001	0.08 ± 0.01	0.01 ± 0.001	0.03 ± 0.002	0.03 ± 0.002	<0.01	ND	ND
			0.05–0.05	0.01–0.01	0.08–0.09	0.01–0.01	0.03–0.04	0.03–0.04			
Rape honeys	5	15	0.21 ± 0.17	0.01 ± 0.00	0.30 ± 0.31	0.06 ± 0.05	ND	0.03 ± 0.006	ND	ND	ND
			0.07–0.44	0.01–0.02	0.11–0.84	0.03–0.15		<0.02–0.04			
Honeydew honeys	6	18	0.52 ± 0.65	0.09 ± 0.03	0.27 ± 0.16	0.35 ± 0.10	ND	0.04 ± 0.03	0.02 ± 0.02	ND	ND
			0.09–1.82	0.03–0.12	0.07–0.37	0.17–0.49		0.01–0.09	<0.01–0.04		
Multifloral honeys	7	21	0.38 ± 0.41	0.02 ± 0.01	0.18 ± 0.22	0.09 ± 0.07	0.04 ± 0.003	0.04 ± 0.02	0.01 ± 0.001	ND	ND
			0.06–1.03	0.01–0.03	0.03–0.63	0.02–0.18	<0.02–0.04	0.01–0.08	<0.01–0.01		
Heather honeys	2	6	0.81 ± 0.12	0.03 ± 0.00	0.14 ± 0.01	0.80 ± 0.38	0.02 ± 0.01	0.07 ± 0.04	ND	ND	ND
			0.72–0.89	0.03–0.03	0.13–0.14	0.53–1.07	<0.02–0.02	0.04–0.10			
Artificial honeys	2	6	0.03 ± 0.00	0.02 ± 0.01	0.06 ± 0.04	0.01 ± 0.01	0.02 ± 0.00	ND	ND	ND	ND
			0.03–0.03	0.01–0.02	0.03–0.08	0.01–0.02	0.02–0.02				
Syrup-feed honeys											
Aloe syrup-feed honey	1	3	0.14 ± 0.001	0.01 ± 0.001	0.18 ± 0.02	0.06 ± 0.001	ND	ND	ND	ND	ND
			0.14–0.14	0.01–0.01	0.17–0.20	0.06–0.06					
Chokeberry syrup-feed honey	1	3	0.05 ± 0.001	0.01 ± 0.000	0.36 ± 0.02	0.10 ± 0.001	ND	0.02 ± 0.001	0.02 ± 0.001	ND	ND
			0.05–0.05	0.01–0.01	0.34–0.37	0.10–0.10		0.02–0.03	0.02–0.02		
Crataegus syrup-feed honey	1	3	0.06 ± 0.002	0.02 ± 0.001	0.14 ± 0.01	0.06 ± 0.003	ND	ND	ND	ND	ND
			0.06–0.06	0.02–0.02	0.14–0.15	0.06–0.07					
Stinging nettle syrup-feed honey	1	3	0.79 ± 0.01	0.01 ± 0.001	0.41 ± 0.01	0.06 ± 0.003	ND	0.03 ± 0.003	0.01 ± 0.001	ND	ND
			0.78–0.80	0.01–0.01	0.40–0.41	0.05–0.06		0.02–0.03	0.01–0.01		
Pine syrup-feed honey	1	3	0.14 ± 0.004	0.01 ± 0.001	0.23 ± 0.01	0.08 ± 0.003	ND	0.09 ± 0.01	ND	ND	ND
			0.14–0.15	0.01–0.01	0.21–0.23	0.08–0.08		0.08–0.10			
Syrup-feed honey	1	3	0.26 ± 0.01	0.02 ± 0.000	0.46 ± 0.01	0.02 ± 0.001	0.02 ± 0.002	0.04 ± 0.001	ND	ND	ND
			0.25–0.26	0.01–0.02	0.46–0.47	0.02–0.02	0.02–0.02	0.03–0.04			
Honeys with natural additives											
Bee honey with chokeberry	1	3	0.35 ± 0.01	0.02 ± 0.001	0.12 ± 0.01	0.06 ± 0.002	ND	ND	0.01 ± 0.001	ND	ND
			0.34–0.36	0.02–0.02	0.11–0.12	0.06–0.06			0.01–0.01		
Bee honey with cinnamon	1	3	0.35 ± 0.03	0.02 ± 0.002	0.26 ± 0.003	0.25 ± 0.01	ND	ND	ND	ND	ND
			0.33–0.38	0.02–0.03	0.25–0.26	0.24–0.26					
Bee honey with pollen	1	3	0.50 ± 0.01	0.04 ± 0.003	0.27 ± 0.02	0.17 ± 0.01	ND	ND	ND	ND	ND
			0.49–0.51	0.03–0.04	0.24–0.28	0.16–0.17					
Royal jelly in honey	1	3	0.12 ± 0.000	0.02 ± 0.001	0.20 ± 0.003	0.07 ± 0.01	ND	0.03 ± 0.002	0.01 ± 0.000	ND	ND
			0.12–0.12	0.02–0.02	0.20–0.20	0.07–0.08		0.03–0.03	0.01–0.01		
Propolis in honey	1	3	0.82 ± 0.04	0.18 ± 0.02	2.92 ± 0.10	0.43 ± 0.002	ND	ND	0.01 ± 0.001	ND	ND
			0.80–0.87	0.17–0.19	2.83–3.03	0.43–0.43			0.01–0.02		
Acacia honey with almonds^a^	1	3	0.02 ± 0.001	0.01 ± 0.001	0.15 ± 0.01	0.02 ± 0.001	ND	0.09 ± 0.003	ND	ND	ND
			0.02–0.02	0.01–0.01	0.15–0.16	0.02–0.02		0.09–0.09			
Acacia honey with peanuts^a^	1	3	0.12 ± 0.01	0.02 ± 0.002	0.10 ± 0.002	0.02 ± 0.001	ND	0.12 ± 0.01	ND	ND	ND
			0.12–0.13	0.02–0.02	0.10–0.10	0.02–0.02		0.11–0.12			
Bee products											
Propolis	2	6	4.33 ± 0.21	0.46 ± 0.14	26.1 ± 32.2	2.39 ± 0.11	0.67 ± 0.16	0.12 ± 0.01	0.02 ± 0.01	ND	ND
			4.18–4.48	0.36–0.56	3.37–48.9	2.31–2.46	0.56–0.78	0.11–0.12	0.01–0.02		
Bee pollen	4	12	2.90 ± 0.16	0.91 ± 0.17	3.70 ± 0.30	1.90 ± 0.45	0.03 ± 0.01	0.13 ± 0.14	0.03 ± 0.01	ND	ND
			2.70–3.06	0.73–1.12	3.26–3.96	1.40–2.47	0.02–0.03	0.03–0.33	0.02–0.04		
Other											
Almonds^a^	1	3	1.81 ± 0.07	0.73 ± 0.03	1.94 ± 0.02	1.51 ± 0.12	0.03 ± 0.002	ND	0.01 ± 0.001	ND	ND
			1.75–1.89	0.71–0.75	1.92–1.95	1.43–1.60	0.03–0.03		0.01–0.01		
Peanuts^a^	1	3	1.72 ± 0.01	0.44 ± 0.03	0.85 ± 0.07	1.31 ± 0.12	0.02 ± 0.001	ND	0.01 ± 0.001	ND	ND
			1.71–1.73	0.42–0.46	0.81–0.90	1.22–1.39	0.01–0.02		0.01–0.01		

*N* number of products, *n* number of analytical subsamples, *ND* not detected. LOD for Cd = 0.003 mg 100 g^−1^, Pb = 0.01 mg 100 g^−1^, Ni = 0.02 mg 100 g^−1^, Co = 0.01 mg 100 g^−1^, Cr = 0.02 mg 100 g^−1^

^a^Honey and its additives were analyzed separately

The macroelements concentrations in the analyzed samples were quite varied, which might result from diverse geographical origin of samples, with the highest values for K and P (Table 4). Concentration ranges of K in 100 g of honeys and bee products samples were as follows: 16.6–73.6 mg (honeys), 11.1–31.7 mg (syrup-feed honeys), 17.9–54.2 mg (honeys with natural additives), and 49.9–70.0 mg (bee products). Latorre et al. (1999) reported almost four times higher K levels in Galician and sesame honeys, while Al-Khalifa and Al-Arify (1999), Terrab et al. (2003), and Yilmaz and Yavuz (1999) lower ones. Chudzinska and Baralkiewicz (2010) determined much higher K concentrations in honeydew and buckwheat honeys, i.e., 264 and 69.5 mg 100 g^−1^, respectively. Also, Siena honeys contained high concentrations of K, i.e., 14.7–413.6 mg 100 g^−1^ (Pisani et al. 2008). Spanish honeys contained between 63.9 and 184.5 mg K in 100 g (Fernàndez-Torres et al. 2005).

P concentration ranged from 3.57 to 69.6 mg in honeys, 7.14–29.7 mg in syrup-feed honeys, 23.2–108 mg in honeys with natural additives, and 573–659 mg 100 g^−1^ in bee products (Table 4). Much lower values, in comparison to our results, were reported for P by Kunachowicz et al. (2005), Souci et al. (2002), and Terrab et al. (2004). Fernàndez-Torres et al. (2005) determined P in Spanish honeys in the range of 6.38 and 14.3 mg P 100 g^−1^.

The highest Ca level was obtained for bee pollen and propolis, 95.1 and 78.0 mg 100 g^−1^, respectively, whereas the lowest was for syrup-feed honey, i.e., 2.25 mg 100 g^−1^. According to Devillers et al. (2002), average Ca concentration in natural honeys amounted to 2.29 mg 100 g^−1^, whereas Capar and Cunningham (2000), Kanoniuk et al. (2004), Nanda et al. (2003), and Yilmaz and Yavuz (1999) determined this macroelement in the range of 4.1 and 5.88 mg 100 g^−1^. Terrab et al. (2004) reported much higher values for Ca. According to Fernàndez-Torres et al. (2005), Ca concentration in Spanish honeys varied between 11.1 and 25.7 mg 100 g^−1^.

The highest Mg concentration was determined in bee pollen, i.e., 77.4 mg 100 g^−1^. Its levels in all other types of the analyzed samples ranged from 0.28 to 25.3 mg 100 g^−1^ (Table 4). Kunachowicz et al. (2005) determined comparable Mg levels, whereas Rashed and Soltan (2004) reported much higher values for clover honeys, i.e., 24.4 mg 100 g^−1^.

Among natural honeys, the richest source of Na was dandelion (7.41 mg 100 g^−1^), whereas the lowest concentration of this metal was determined in chestnut honey (0.78 mg 100 g^−1^). Syrup-feed honeys and honeys with natural additives characterized by Na levels in the range of 0.59 and 2.43 mg 100 g^−1^ (Table 4). Comparable results for Na are reported by Capar and Cunningham (2000), Conti (2000), Latorre et al. (1999) as well as Souci et al. (2002). However, Latorre et al. (1999), Nanda et al. (2003), Terrab et al. (2004), and Yilmaz and Yavuz (1999) found higher concentrations in natural bee honeys.

Zinc concentration in the honeys samples analyzed ranged between 0.02 and 1.82 mg 100 g^−1^ (Table 5). The average concentrations of Zn were the highest in bee products such as propolis (4.33 mg 100 g^−1^) and bee pollen (2.90 mg 100 g^−1^), while the lowest were in acacia honey with almonds (0.02 mg 100 g^−1^). According to Souci et al. (2002) and Chudzinska and Baralkiewicz (2010), average Zn level in honeys amounted to 0.35 and 0.32 mg 100 g^−1^, respectively. The latter was conducted on 55 honey samples, which consisted of three different types of honey: honeydew, buckwheat, and rape honey. Comparable results for Zn are reported by Devillers et al. (2002), Kump et al. (1996), Latorre et al. (1999), Terrab et al. (2003), Tuzen et al. (2007), and Yilmaz and Yavuz (1999).

The lowest mean Cu concentration was determined in acacia, orange, and rape honeys—0.01 mg 100 g^−1^ (Table 5). The mean Cu values were significantly higher in apple honeys, propolis in honey, propolis, and bee pollen samples amounting to 0.22, 0.18, 0.46, and 0.91 mg 100 g^−1^, respectively. Chudzinska and Baralkiewicz (2010) reported Cu concentration in buckwheat honeys in the range of 0.03 and 0.16 mg 100 g^−1^. According to Szefer and Grembecka (2007), Cu levels in honeys were in the range of <0.005 (acacia and Galician honeys) and 0.18 mg 100 g^−1^ (orange and sesame honeys). Tuzen et al. (2007) determined Cu in the range of 0.02–0.24 mg 100 g^−1^ in multifloral honey samples from different regions of Turkey.

Fe levels in the samples analyzed ranged from 0.03 to 48.9 mg 100 g^−1^ (Table 5). The highest Fe average content was obtained for propolis (26.1 mg 100 g^−1^). Lower values of Fe than these obtained in this study are reported by Souci et al. (2002) and Kunachowicz et al. (2005). While Devillers et al. (2002) determined comparable levels of Fe, Kump et al. (1996) determined higher ones, i.e., 0.76 mg 100 g^−1^. Fe concentrations in honeys are comparable with those observed by Conti (2000); Latorre et al. (1999) and Terrab et al. (2003). Turkish honeys contained Fe in the range of 0.18–1.02 mg (Tuzen et al. 2007).

Also in the case of Mn, its average concentration was found to be the highest in propolis samples, i.e., 2.39 mg 100 g^−1^. Mn concentrations in honeys are comparable with those observed by Conti (2000), Latorre et al. (1999), and Terrab et al. (2003). Al-Khalifa and Al-Arify (1999) determined lower levels of this element in honeys, i.e., 0.01 mg 100 g^−1^.

Chromium content in the samples analyzed ranged from <0.02 to 0.67 mg 100 g^−1^ (Table 5). Higher Cr concentrations were determined in honeys by Devillers et al. (2002) and Kump et al. (1996), while lower concentrations were determined by Caroli et al. (1999). According to Souci et al. (2002), Cr concentration in honeys amounted to 0.013 mg 100 g^−1^.

The highest Ni and Co concentrations were found in bee pollen samples (0.13 and 0.03 mg 100 g^−1^, respectively). Buldini et al. (2001), Devillers et al. (2002), and Latorre et al. (1999) determined comparable levels of Ni and Co. However, Rashed and Soltan (2004) reported higher levels of both elements Ni and Co.

Consumption of 25 g of honeys and bee products supplies the human body with varied amounts of mineral components. In general, the realization of RDA for an adult by 25 g of all the analyzed honeys (natural, syrup-feed, and with additives) was between 0.22 % and 0.30 % for Mg, 0.17 % for Ca, 0.03 % for Na, 0.17 % for K, 1.28 % for P, 0.84–1.15 % for Zn, 1.03 % for Cu, 0.61–1.10 % for Fe, and 1.65–2.11 % for Mn. The highest average percentages of realization of RDA for adult (Jarosz and Bułhak-Jachymczyk 2008) were obtained for bee products, i.e., 3.58–4.83 % for Mg, 2.24 % for Ca, 0.12 % for Na, 0.34 % for K, 22.5 % for P, 7.68–10.6 % for Zn, 21.1 % for Cu, 15.5–28 % for Fe, and 22.4–28.5 % for Mn.

Levels of toxic elements in all the samples analyzed were <45 μg 100 g^−1^ and <15 μg 100 g^−1^ for Pb and Cd, respectively, it means that due to Cd intake with the analyzed products PTMI is not exceeded. Therefore, it was concluded that there is no health hazard associated with consumption of honeys and bee products.

### Statistical estimate

#### Correlation

The analyzed samples before Spearman’s rank correlation analysis were divided into three groups, i.e., natural honeys, syrup-feed honeys, and honeys with natural additives and bee products. The majority of chemical elements exhibited significant positive and negative correlations between their concentrations in honeys and bee products samples. The most significant relationships in natural honeys samples were noted for the following pairs of elements: Mg–K–Cu–Mn (*p* < 0.001), Na–K (*p* < 0.001), P–Cu–Mn (*p* < 0.001), Zn–Fe (*p* < 0.01), Na–Cu, Mn–Cu, Mg–P, K–P, and Mn–Na (*p* < 0.05). Significant negative correlations (*p* < 0.05) were observed between the concentrations of Cu and Na in syrup-feed honeys. In the case of honeys with natural additives, significant positive relationships were observed for the following assemblages: Na–Zn, K–Zn (*p* < 0.01), Na–Cu–Fe–K, Cu–P, Zn–Cu, and Fe–Mn (*p* < 0.05). Significant positive relationships were also observed in the group of bee products between such elements as Mg and K (*p* < 0.001), Ca–Cu (*p* < 0.01), Ca–Mn, and Na–Cu (*p* < 0.05).

#### ANOVA Kruskal–Wallis test

The influence of the type, and botanical and geographical provenance on the products’ elemental composition was verified by Kruskal–Wallis test. There was a statistically significant influence of botanical origin of honey (acacia, buckwheat, lime, rape, honeydew, multifloral, heather) on K, P, Cu, and Mn (*p* < 0.001), Mg (*p* < 0.01), and Ca (*p* < 0.05). The geographical provenance of the product had an influence on Zn content in honey samples (*p* < 0.01). Levels of some of the analyzed elements in honeys strongly depended on their type (natural honeys, syrup-feed honeys, and honeys with natural additives). Such interdependences were observed in the case of Ca, Mg (*p* < 0.01), and Cu (*p* < 0.05).

#### Factor analysis

**Fig. 1:**
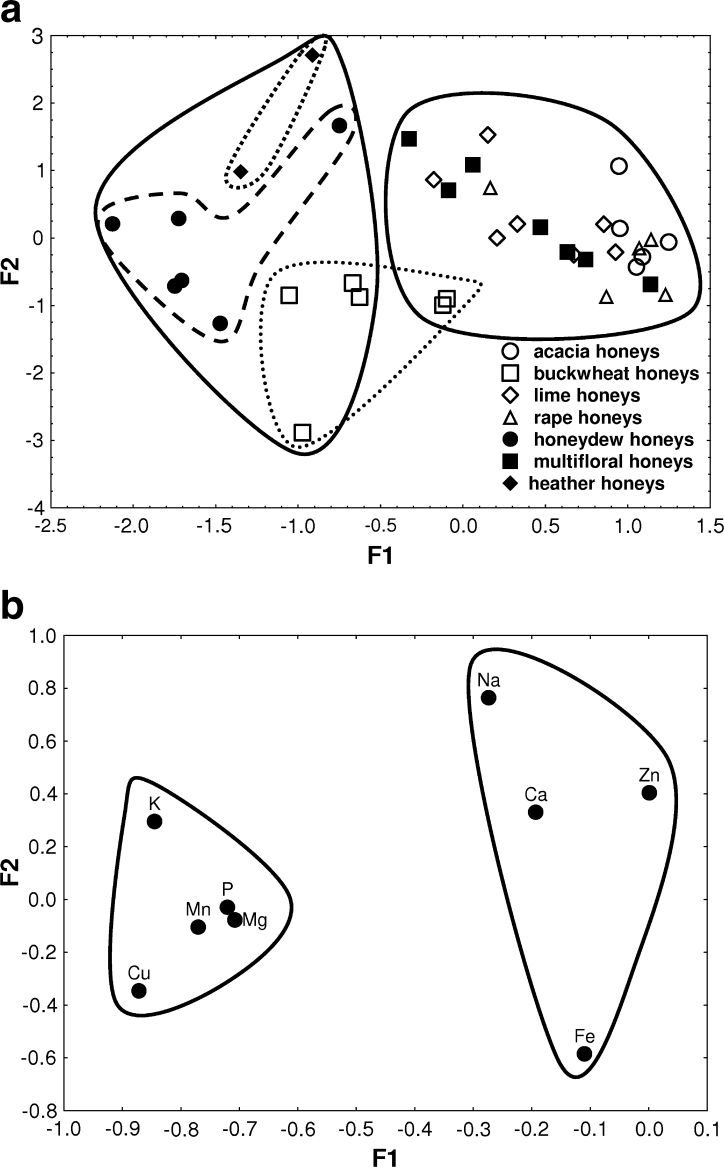
**a** Scatterplot of object scores of the two discriminant functions of exclusively natural honeys. **b** Scatterplot of loadings for nine elements in natural honeys samples

Application of FA model to data from multi-elemental analysis of natural honeys samples indicated botanical differences between them. The results for honeys data set are presented in Fig. 1a and b. The first two factors explain cumulatively 51.5 % of the total variance; F1 and F2 account for 35.7 and 15.8 %, respectively. The eigenvalues are 3.21 (F1) and 1.42 (F2), respectively. Factor loadings for the elements analyzed are presented in Table 2. Dark color honeys such as honeydew, buckwheat, and heather are generally characterized by lower values of F1, whereas the light color ones, i.e., acacia, lime, rape, and multifloral, by its higher values (Fig. 1a). Thus, factor F1 can be interpreted as a factor distinguishing dark color honeys from light ones. F1 achieves the lowest values for K, Cu, Mn, P, and Mg (as descriptors for dark color honeys), while the highest for Zn, Fe, Ca, and Na (as descriptors for light color honeys) (Fig. 1b). The lowest F2 values correspond to objects representing buckwheat honey samples that are rich in Cu and Fe. Higher values of this factor can be associated with heather honeys, which contain greater amounts of Na.

**Fig. 2:**
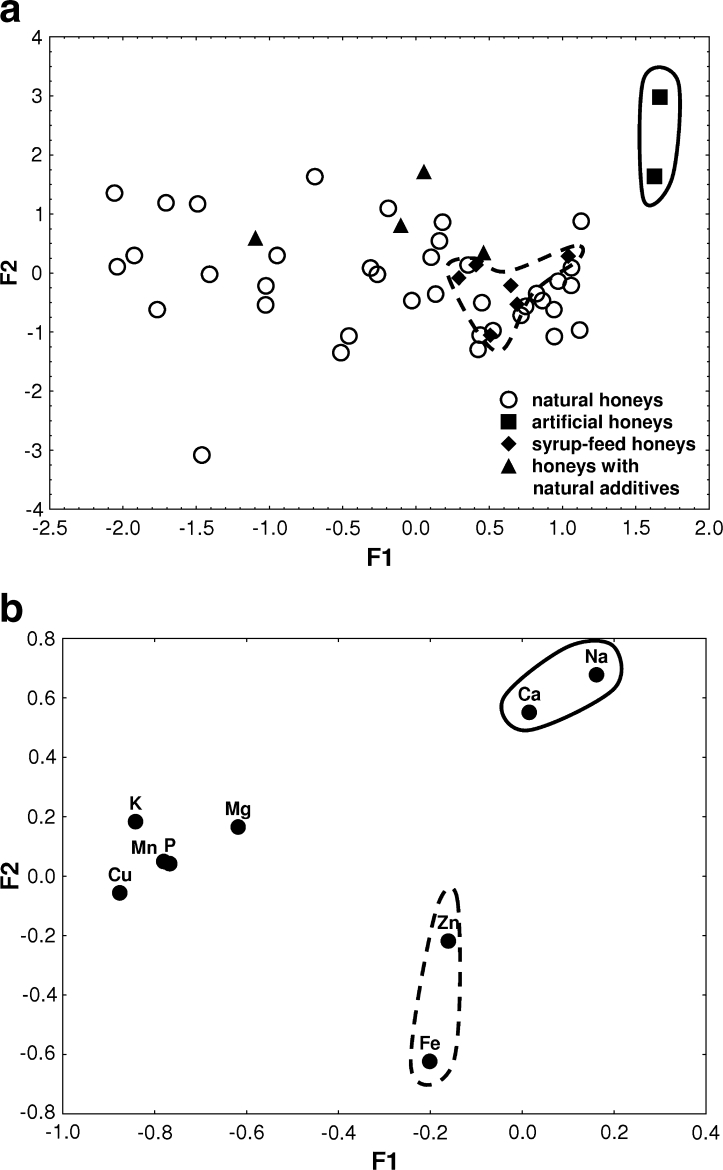
**a** Scatterplot of object scores of the two discriminant functions of natural, syrup-feed, artificial, and with natural additives honeys. **b** Scatterplot of loadings for nine elements in analyzed samples

In order to visualize the data structure concerning natural, syrup-feed, artificial, and with natural additives honeys, a factor analysis was carried out and the results depicted in Fig. 2a and b. The two factors (F1, F2) issued from factor analysis explain cumulatively up to 49.1 % of the total variance, so that 35.0 % is explained by F1 and 14.1 % by F2. The eigenvalues are 3.15 (F1) and 1.27 (F2), respectively. Factor loadings for the elements analyzed are presented in Table 3. Figure 2a shows the scatterplot for the studied samples. In order to identify elements responsible for the grouping of the objects (honeys), biplot of loadings was drawn for F1–F2 (Fig. 2b). As can be seen in Fig. 2, higher values of F1 and F2 correspond to artificial honey samples characterized by the highest levels of Ca and Na. It means that Ca and Na are the best descriptors for identification of artificial honeys (Fig. 2b). The lowest F1 values can be associated with natural honeys and those with natural additives described by K, P, Cu, Mn, and Mg (Fig. 2a, b). The distribution of the points corresponding to the individual elements shows that factor F2 achieves the lowest values for natural and syrup-feed honeys containing great concentration of Fe and Zn (Fig. 2a, b).

#### Cluster analysis

**Fig. 3:**
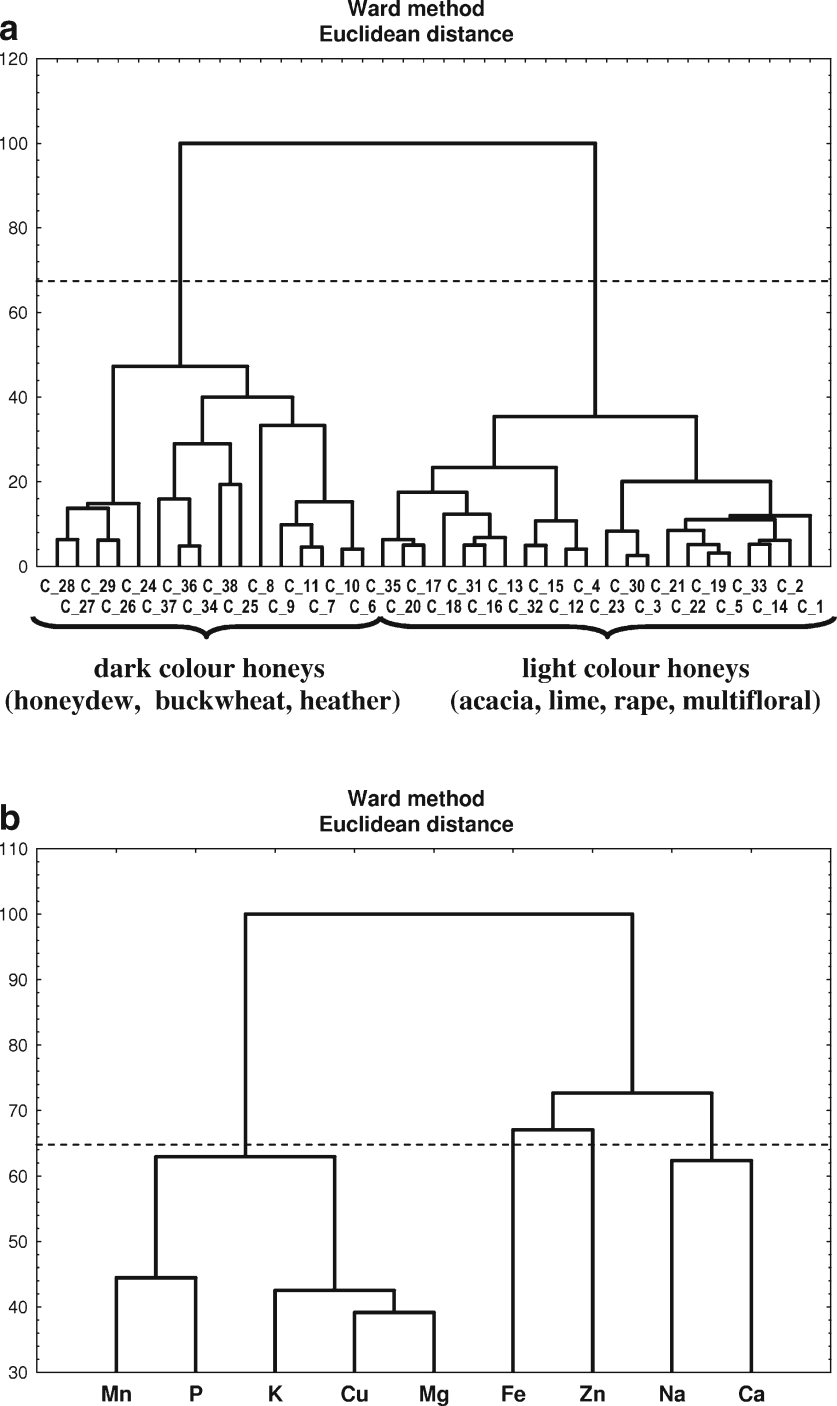
**a** Hierarchical dendrogram for 38 natural honeys samples as objects. **b** Hierarchical dendrogram for nine elements as objects

Cluster analysis (CA) was applied in order to identify interrelationships between concentration of selected elements in the studied material on one hand and belonging of honeys to a particular group on the other hand. The numbers of significant clusters in the dendrograms were based on the Sneath index, using 66 % of maximum distance measure. The CA data (hierarchical clustering, Ward’s method) for natural honeys as objects is shown in Fig. 3a and b. The dendrogram is built up of two main clusters. The first contains objects, which represents dark color honeys, whereas the latter light color ones. It is also possible to distinguish two subclusters in both of the clusters. The dark color honeys cluster (except C34 and C36) contains samples representing honeydew (C24–C29), buckwheat (C6–C11), and heather honeys (C37, C38), while the second cluster consists of acacia (C1–C5), lime (C12–C18), rape (C19–C23), and multifloral honeys (C30–C36). The obtained information confirms the results of FA analysis for the matrix of natural honeys samples.

**Fig. 4:**
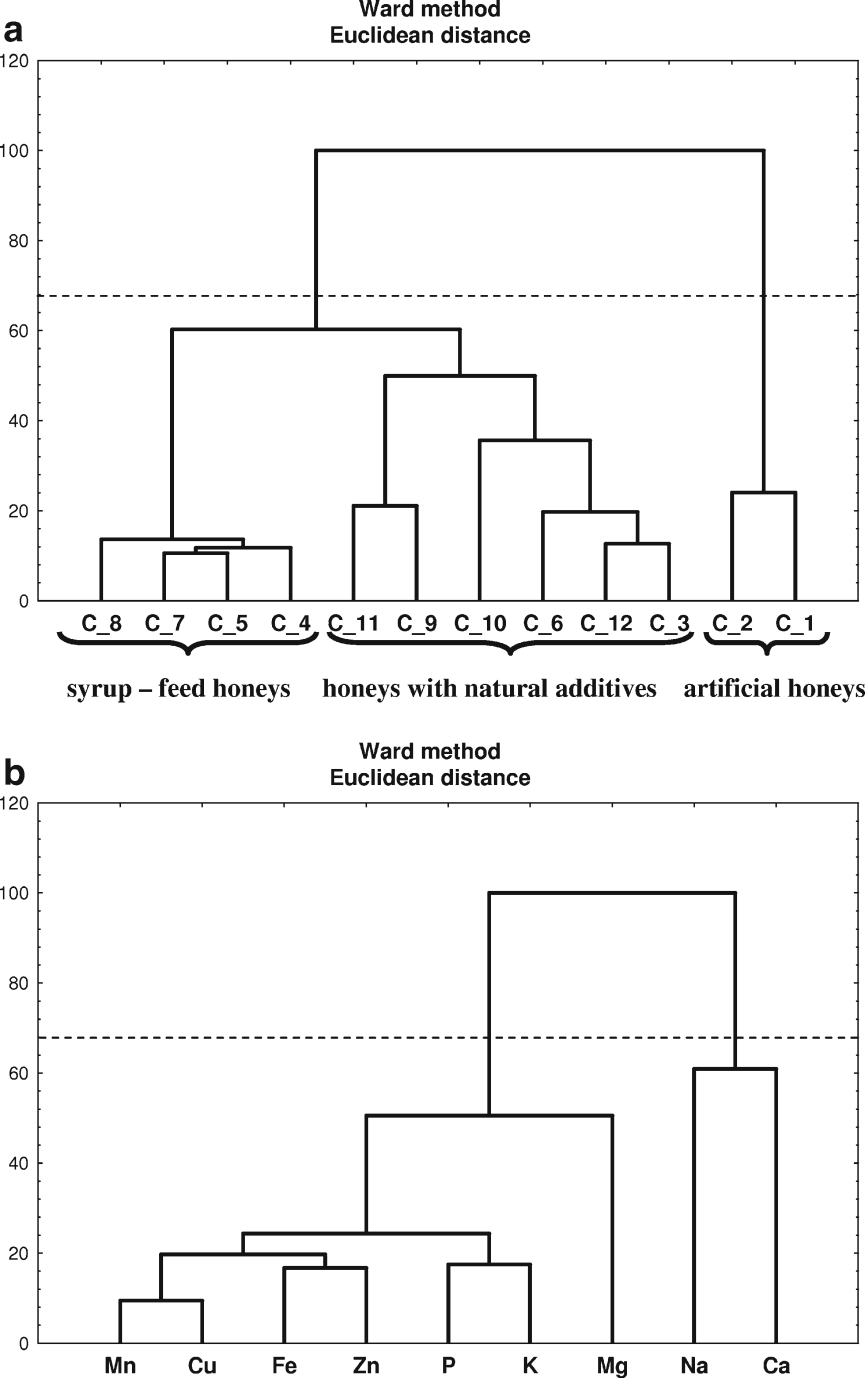
**a** Hierarchical dendrogram for syrup-feed, artificial, and with natural additives honeys as objects. **b** Hierarchical dendrogram for nine elements as objects

Hierarchical dendrogram for the analyzed samples of syrup-feed and with natural additives honeys and artificial ones as objects is depicted in Fig. 4a and b. There can be distinguished two main clusters, the first one (C1–C2) contains objects representing artificial honeys while the second syrup-feed and with natural additives honeys. As can be observed, artificial honeys are distinguished by two metals, i.e., Na and Ca (Fig. 4b), which was also confirmed by FA analysis. Syrup-feed honeys were generally assigned to one subcluster (C4, C5 and C7, C8) except for the samples of stinging-nettle and aloe syrup-feed honeys (C3 and C6), which can be found in the subcluster of honeys with natural additives (C9–C12). These results have shown that there is a possibility of CA application in fraud detection as artificial honeys are well distinguished from other samples. What is more, it can be concluded that this technique is able, based on mineral composition, to distinguish samples not only of varied type but also in view of their botanical provenance as well as level of technological processing.

## Conclusions

According to the results obtained, honeys and bee products proved to be products that might not only provide significant amounts of energy but also of essential nutrients as Mg, K, Ca, Zn, Cu, Fe, and Mn. Generally, darker honeys had a higher mineral content than the light color ones. However, it must be also remembered that the contribution of honey to the recommended daily intake (RDI) is small, but its importance lies in its physiological effects. Based on RDA estimated for essential elements, it was concluded that bee products such as bee pollen and propolis supply an organism with the biggest amounts of bioelements. In the light of PTMI estimated for toxic elements, there is no health hazard associated with exposure to Cd and Pb through the consumption of these products.

Application of ANOVA Kruskal–Wallis test let us reveal a relationship between honey biological provenance and its elemental composition. Macro- and microelements levels in the analyzed samples were significantly influenced by the extent of technological processing of samples as well as geographical origin.

Other chemometric techniques such as factor and cluster analyses have proved to be reliable tools in the differentiation of food products in view of their mineral composition. Their application was helpful for a deeper understanding of the distribution of selected metals in food. Moreover, these techniques let us clearly separate artificial honey samples from the natural ones, which might be very helpful in detecting fraud or proving authenticity of the product. Based on the obtained results, it can be concluded that multivariate techniques are efficient tools that can be successfully applied to food quality and authenticity evaluation.
